# Congenital hypothyroidism due to ectopic sublingual thyroid gland in Prader-Willi Syndrome: a case report

**DOI:** 10.1186/s13052-017-0403-7

**Published:** 2017-09-22

**Authors:** Sarah Bocchini, Danilo Fintini, Graziano Grugni, Arianna Boiani, Alessio Convertino, Antonino Crinò

**Affiliations:** 1 Autoimmune Endocrine Diseases Unit, Bambino Gesù Children’s Hospital, Research Institute, Palidoro Rome, Italy; 2 Endocrinology and Diabetology Unit, Bambino Gesù Children’s Hospital, Research Institute, Palidoro Rome, Italy; 3 Division of Auxology, Italian Auxological Institute, Research Institute, Verbania, Piancavallo Italy

**Keywords:** Prader-Willi syndrome, Hypothyroidism, Obesity, Hypotonia, Obesity

## Abstract

**Background:**

Thyroid gland disorders are variably associated with Prader-Willi syndrome (PWS).

Many of the clinical features in newborns with PWS are similar to those found in congenital hypothyroidism (CH).

**Case presentation:**

We report a case of a girl with CH and PWS. At the age of 9 months CH caused by an ectopic sublingual thyroid was diagnosed, and hormone replacement therapy was started. In spite of this treatment a decrease in growth velocity, weight excess and delayed development were observed. At the age of 9 years PWS was suspected on the basis of phenotype and genetic tests confirmed a maternal uniparental disomy of chromosome 15. This is the second reported case of hypothyroidism due to an ectopic sublingual thyroid gland in PWS suggesting that, although rare, an association between CH and PWS may exist. In our case diagnosis of PWS was delayed because mental retardation, hypotonia, obesity and short stature were initially attributed to hypothyroidism.

**Conclusions:**

In this context PWS should be considered in obese children with CH who do not improve adequately with l-thyroxine therapy. Also, thyroid function in all PWS children should be assessed regularly in order to avoid delayed diagnosis of hypothyroidism.

## Estabilished facts

- Thyroid axis dysfunction does not seem to be a very common feature during infancy in PWS.
- Many of the clinical features in newborns with PWS are similar to those found in congenital hypothyroidism.

## Novel insights

- The presence of congenital hypothyroidism may delay the diagnosis in subjects affected by PWS.
- PWS should always be considered in patients with congenital hypothyroidism and failure to thrive, and who do not improve adequately with the thyroid hormone replacement therapy.

## Background

Prader-Willi syndrome (PWS) is a rare genetic disorder caused by the absent expression of the paternal active genes in the PWS critical region of chromosome 15. Three main genetic mechanisms are responsible for PWS: paternal chromosome 15q11-q13 deletion (70%), maternal uniparental disomy for chromosome 15 (UPD15) (20–30%), and imprinting center defects (ID) (1–3%) [1]. The birth prevalence of PWS with molecular diagnosis is estimated to be about 1:15,000 [2], representing the most common syndromal cause of life-threatening obesity [3]. The clinical phenotype of PWS patients is mainly characterized by neonatal hypotonia, hyperphagia leading to severe obesity in early childhood (if uncontrolled), short stature, multiple endocrine defects, small hands and feet, scoliosis, sleep disorders, developmental delay with cognitive impairment, behavioural and learning problems and characteristic facial appearance [3, 4].

A complex hypothalamic dysfunction is believed to be responsible for this multifaceted phenotype, including a dysregulation of GH-IGF-I axis, hypogonadism, adrenal insufficiency, and altered pituitary-thyroid axis [5, 6].

Published data on disorders of thyroid function and morphology in PWS are very limited. Thyroid axis dysfunction is not very common during infancy in PWS, while hypothyroidism variably affects a significant number of older children and adults with PWS. Untreated thyroid axis impairment causes the most damaging consequences during the first months of infancy, when thyroid hormones exert a critical action on neurological development. In this context, a recent study revealed a normal neonatal thyroid screening test in 23 newborns with PWS [7]. On the other hand, an investigation during the first 2 months of postnatal life found low free thyroxine (FT4) levels, in the presence of normal thyroid-stimulating hormone (TSH) in one third of subjects with PWS [8]. Moreover, other authors have demonstrated that a thyrotropin-releasing hormone (TRH)-TSH thyroid axis dysfunction is a common feature in infants with PWS [9]. It is of note that hypothyroidism, similarly to PWS, is characterized by hypotonia and delayed psychomotor development when present early in life, and is associated with weight gain, impaired growth and learning problems when it is present during childhood [10, 11].

In addition to the disruption of the thyroid function at the central level [12], other causes of neonatal thyroid abnormalities have been reported in PWS subjects, including the presence of fetal goiter in a newborn with UPD15 [13] and ectopic location of the thyroid gland in 1-yr-old female with CH [14].

We report the second case of sublingual thyroid gland in a female patient with PWS, where the diagnosis of congenital hypothyroidism was missed on newborn screening.

## Case presentation

**Fig. 1:**
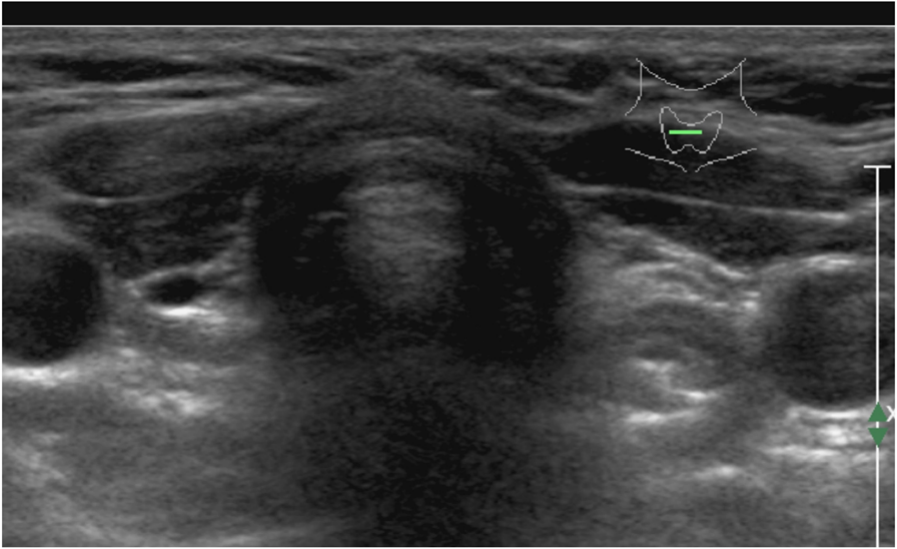
Ultrasonography of the neck showed absence of thyroid tissue

**Fig. 2:**
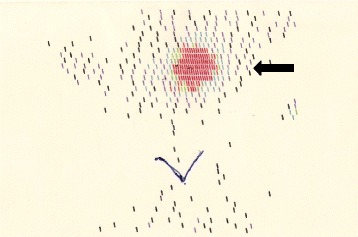
99mTc-pertechnetate thyroid scintigraphy showed a small, ectopic, sublingual thyroid gland (black arrow)

The patient is a 28.9-year-old woman, born after a full-term pregnancy from unrelated healthy parents. Maternal age was 44 years and paternal age was 40. Foetal movements were decreased during pregnancy. Caesarean section was necessary because of premature rupture of the membranes. Birth weight was 2450 g. After delivery the patient showed severe muscular hypotonia, weak cry, sleepiness, poor deep tendon reflexes and abnormalities in thermoregulation. Feeding problems were noted due to a poor sucking reflex and the baby required gavage. She was discharged with diagnosis of congenital benign hypotonia. She had a psychomotor delay, non-specific mild dysmorphisms and strabismus. Karyotype was normal (46, XX). Neonatal screening for CH showed normal thyroxine (T4) levels and a slightly elevated TSH (24 μU/ml), both of which were confirmed with a blood sample. Nevertheless, she was not treated. The patient was firstly admitted at our hospital at 9 months of age because of elevated serum level of TSH (40 μU/ml - nv <4 μU/ml), with normal FT4 (8 pg/ml – n.v. 5–12.5 pg/ml). She had a supine length of 66 cm *(<10th percentile)*, a body weight of 8 kg *(<25th percentile)* and a head circumference of 45.5 cm *(97th percentile)*. The thyroid ultrasound revealed the absence of the gland in the neck (Fig. 1), while the 99mTc-pertechnetate thyroid scintigraphy showed a small, ectopic, sublingual thyroid gland (Fig. 2).

Thus, levo-thyroxine therapy was promptly initiated (8 μg/kg/die) and the dose was gradually adjusted over several months with normalization of FT4 and TSH levels.

Due to the persistence of hypotonia and poor response to stimuli, associated with the absence of sucking reflex in the first weeks of life, a brain computed tomography scan was done at 12 months of age, which showed ventricular enlargement and craniosynostosis.

Two years later, at the age of three, the patient showed poor physical and developmental progress, despite being biochemically euthyroid on levo-thyroxine substitution therapy, and associated hyperphagia, weight gain, short stature and decreased growth velocity. In addition, physical examination revealed a more distinctive phenotype, characterized by peculiar facial features (narrow bifrontal diameter, dolichocephaly, almond-shaped eyes, downturned angles of the mouth) with abundant thick saliva, small hands and feet. At this age a severe scoliosis was diagnosed, treated initially with a corset. The patient later required surgery at the age of 13.

To evaluate growth retardation, two growth hormone (GH) stimulation tests (clonidine 0.150 mg/m^2^ orally and insulin tolerance test 0.1 UI/kg i.v. bolus) were performed and both of them were consistent with a diagnosis of GH deficiency [GH peak 1.7 ng/ml and 5.6 ng/ml, respectively (nv >10 ng/ml)]. The combined test [GHRH (1 μg/kg) + Arginine (0,5 g/kg iv)] confirmed the presence of reduced GH response [GH peak 8.7 ng/ml (nv >20 ng/ml)]. Pituitary MRI revealed an empty sella with a small, hypoplastic pituitary gland at its base.

Therefore, GH therapy was initiated at 0.24 mg/kg/week, subsequently dose adjusted based on IGF-1 levels and growth curve, and continued until the age of 12.9 years.

At the age of 9, given her constellation of signs and symptoms, PWS diagnosis was considered, as a possible cause of her clinical picture, but because of the mother’s initial refusal genetic tests were performed only at the age of 16. Methylation analysis and DNA polymorphism analysis of chromosome 15 confirmed the presence of UPD15.

Since the age of 10.2 both central and obstructive sleep apnea were noted on polysomnographic study, for which the child subsequently required non-invasive ventilation (NIV).

She also presented skin-picking starting at age 11 and worsening with age.

At the age of 14.1 a right ovarian cyst was removed. Since our patient had no spontaneous menarche, she underwent a LHRH test at age 22 that documented a complete hypogonadism of central origin with concomitant low estradiol levels. Consequently, sex steroid replacement was initiated.

Corticotrophin deficiency was excluded because cortisol response to low-dose ACTH test was normal (22 μg/dL - nv >18.1 μg/dL).

Starting from transition phase, she had episodes of psychosis that required antipsychotic therapy. Over the years a progressive worsening of obesity was observed, due to her uncontrolled eating habits and poor compliance with diet therapy. At the age of 24 a bioenteric intragastric balloon (BIB) was inserted for treatment of morbid obesity, with a transient weight loss of 13 kg in 9 months.

At the last examination (28.9 years), the patient’s anthropometric data were the following: height 142.8 cm (−3.07 SDS); weight 119.2 kg, body mass index (BMI) 59 kg/m^2^. Blood pressure was normal. She presented mild mental retardation, outcomes of operated scoliosis and skin-picking, and continued utilization of NIV for sleep disordered breathing. Oral glucose tolerance test was normal, but hyperinsulinism was present. She is on therapy with L-thyroxine (125 μg/die), sex steroids replacement and psychotropic drugs (risperidone and carbamazepine) for recurrent psychotic crises.

## Discussion

Up until the last decade the thyroid axis of PWS subjects was generally considered to be normal or slightly altered, with a similar frequency of hypothyroidism in comparison to the general population [15–17]. More recently, other studies reported a higher prevalence of hypothyroidism in PWS, mostly due to a central defect of central origin (hypothalamic hypothyroidism) [8, 9, 12, 18–20].

At present, data on the incidence of other thyroid alterations, including primary hypothyroidism, are inconsistent in these patients. In this light, the most interesting finding in our PWS patient is her concurrent CH, due to a sublingual ectopic thyroid gland. To our knowledge, this is the second reported occurrence of ectopic thyroid tissue in PWS [14]. In our case, high levels of TSH with normal T4 (subclinical hypothyroidism) was detected during the neonatal screening for CH, but were not considered by the neonatologist (the child was born in a small provincial hospital) and levo-thyroxine therapy was started only at the age of 9 months. In this regard, our clinical case reports a dramatic management error of a newborn, who was screened for CH but was not treated promptly. However, difficulties in the interpretation of thyroid status may be explained by only slightly elevated TSH levels, probably due to combined peripheral and central hypothyroidism, as suggested by the later findings of GH deficiency, central hypogonadism and empty sella on MRI.

Furthermore, our case is unique in that the diagnosis of PWS was delayed because mental retardation, hypotonia, weight excess and short stature were initially attributed to hypothyroidism.

In this regard, the majority of newborns with PWS show signs and symptoms which can simulate CH, e.g. muscular hypotonia, lethargy and poor sucking often resulting in failure to thrive. Consequently, pediatricians should be aware that patients who are found to have CH and severe infantile hypotonia and do not improve with adequate thyroid replacement therapy need to be further investigated for an additional diagnosis, including genetic evaluation for PWS, particularly when a distinctive phenotype becomes evident.

Finally, it is of note that a sublingual ectopic thyroid may induce local symptoms such as dysphagia, related to the potential growth of the thyroid tissue [21]. Careful consideration should be made about dysphagia in children with PWS, because swallowing dysfunction and choking may be a contributor to morbidity and mortality in PWS.

Recently, oropharyngeal phase dysphagia with abnormal pharyngeal clearance have been demonstrated by videofluoroscopic swallow studies in infants with PWS [22].

## Conclusions

In conclusion, this report suggests that, although rare, the association between CH and PWS may exist. Because of overlapping symptoms and signs the presence of CH may delay diagnosis of PWS. In this light, PWS should always be considered in patients with CH and failure to thrive who do not improve adequately with levo-thyroxine therapy.

As a consequence, current clinical management of both pathological conditions should be revised in order to avoid diagnostic delay. In this light, it might be advisable to regularly assess thyroid function in all PWS children, even if neonatal screening for CH is negative.

## Abbreviations

PWS: Prader-Willi syndrome
CH: Congenital hypothyroidism
UPD15: Maternal uniparental disomy for chromosome 15
ID: Imprinting center defects
GH: Growth hormone
TSH: Thyroid-stimulating hormone
FT4: Free thyroxine
TRH: Thyrotropin-releasing hormone
NIV: Non-invasive ventilation
